# Instruments for the detection of frailty syndrome in older adults: A systematic review

**DOI:** 10.1371/journal.pone.0216166

**Published:** 2019-04-29

**Authors:** Jossiana Wilke Faller, David do Nascimento Pereira, Suzana de Souza, Fernando Kenji Nampo, Fabiana de Souza Orlandi, Silvia Matumoto

**Affiliations:** 1 Department of Maternal and Child Health and Public Health, University of São Paulo, PAHO/WHO Collaborating Center for Nursing Research Development, Ribeirão Preto School of Nursing, Ribeirão Preto, Brazil; 2 Program in Health Promotion and Care in Hospital Care of the Medical School of the University of São Paulo, São Paulo, Brazil; 3 Latin-American Institute of Life and Natural Sciences, Federal University of Latin-American Integration, Foz do Iguassu, Paraná, Brazil; 4 Department of Gerontology of the Federal University of São Carlos, São Carlos, São Paulo, Brazil; Cardiff University, UNITED KINGDOM

## Abstract

Frailty is a dynamic process in which there is a reduction in the physical, psychological and/or social function associated with aging. The aim of this study was to identify instruments for the detection of frailty in older adults, characterizing their components, application scenarios, ability to identify pre-frailty and clinimetric properties evaluated. The study was conducted according to the Preferred Reporting Items for Systematic Reviews and Meta-Analyses (PRISMA), under registration number CRD42017039318. A total of 14 electronic sources were searched to identify studies that investigated instruments for the detection of frailty or that presented the construction and/or clinimetric evaluation of the instrument, according to criteria established by the COnsensus-based Standards for the selection of health Measurement INstruments (COSMIN). 96 studies were included in the qualitative synthesis: 51 instruments for the detection of frailty were identified, with predominantly physical domains; 40 were constructed and/or validated for use in the older adult community population, 28 only highlighted the distinction between frail and non-frail individuals and 23 presented three or more levels of frailty. The FRAGIRE, FRAIL Scale, Edmonton Frail Scale and IVCF-20 instruments were the most frequently analyzed in relation to clinimetric properties. It was concluded that: (I) there is a large number of instruments for measuring the same construct, which makes it difficult for researchers and clinicians to choose the most appropriate; (II) the FRAGIRE and CFAI stand out due to their multidimensional aspects, including an environmental assessment; however, (III) the need for standardization of the scales was identified, since the use of different instruments in clinical trials may prevent the comparability of the results in systematic reviews and; (IV) considering the different instruments identified in this review, the choice of researchers/clinicians should be guided by the issues related to the translation and validation for their location and the suitability for their context.

## Introduction

Frailty is a dynamic process in which there is a reduction in the physical, psychological and/or social functions, associated with aging and detrimental to the health. This condition represents a potential public health problem due to the multiple clinical and social consequences and its dynamic nature [1]. Identifying frail older adults or those at risk of frailty should be one of the foundations of geriatric care, since it is a complex and important issue associated with aging, with implications for both the patients and the use of the health services [2]. Adequate recognition of frailty may reduce risks from possibly detrimental interventions, with it being unacceptable to consider patients only on the basis of chronological age [3]. The dynamic nature of frailty highlights a potential for preventive and restorative interventions [2], so that when detected early, it is possible to preserve the functional and cognitive reserves, to maintain the capacity for self-care and to prevent disabilities, falls, functional decline, institutionalization, hospitalization and death.

Approximately 10% of people over 65 and 25% to 50% of those over 85 are frail, according to the criteria established by Fried [4]. In the countries of Latin America and the Caribbean (LAC), with a high prevalence of chronic and incapacitating diseases, one in five older adults are considered frail [5]. However, according to the definition of frailty, the criteria for inclusion or exclusion of the population in the studies and the diagnostic parameters used in the clinical practice and in epidemiological studies, the reported prevalence rates of frailty vary substantially from 4.0% to 59.1%. This variation was identified in 21 studies, with a mean prevalence of 10.7% (95% CI: 10.5–10.9%), with the highest frequencies observed in studies that used multidimensional instruments to evaluate this construct [6].

There are a growing number of instruments that aim to evaluate frailty; however, researcher must be attentive to the choice of the most appropriate and precise in order to guarantee the quality of their results. The data must be accurate, valid and interpretable for the health assessment of the population, as well as providing scientifically sound results. The performance of the results of these measures depends to a large extent on the reliability and validity of the instruments [7]. Thus, the selection of a measuring instrument with inadequate clinimetric properties may cause bias in the conclusions of the studies, wasting resources, increasing costs and risking the participants and/or population [8].

Before starting this study, the literature was searched for systematic reviews addressing frailty in older adults. The reviews on validation of frailty evaluation instruments [2, 9–15] focused on identifying the clinical definition of frailty and the instruments for its evaluation [10]; the accuracy of the diagnostic tests [13]; the score system of the instruments in relation to values predictive of frailty [12]; instruments and their clinimetric properties [2, 9, 14]; frailty screening instruments specifically for use in primary healthcare [11]; and the systematic categorization of the instruments and contexts of use [15]. Although three reviews addressed validation aspects [2, 9, 14], only two [2, 9] substantially explored the validity aspects of the instruments.

Given the vast expansion in the literature on frailty, the increase in the world’s older adult population, the prevalence of frailty in this population and the adverse events due to this syndrome, identifying instruments consistent with the multifactorial and complex nature of the syndrome remains a priority for use in both clinical trials as well as the clinical practice. Thus, this study aimed to identify instruments for the detection of frailty syndrome in older adults, characterizing them according to their components, application scenarios and ability to identify pre-frailty, as well as to present the clinimetric properties evaluated: validity, reliability, sensitivity, specificity, positive predictive value and negative predictive value.

## Methods

This systematic review was conducted in accordance with the Preferred Reporting Items for Systematic Reviews and Meta-Analyzes (PRISMA) [16]. The steps followed in preparing this review were: 1. Elaboration of the research question; 2. Elaboration of the protocol and registration in the International prospective register of systematic reviews (PROSPERO), under number CRD42017039318; 3. Execution of the searches in the databases; 4. Selection of studies according to the eligibility criteria; 5. Extraction of data from the primary studies and; 6. Synthesis of results [16, 17].

The electronic searches were performed on April 18, 2017 and updated on September 25, 2018. The electronic databases searched were the Medical Literature Analysis and Retrieval System Online (MEDLINE), EMBASE, Scopus, Ovid, ProQuest, Web of Science, Latin American and Caribbean Health Sciences Literature (LILACS), Pan American Health Organization (PAHO), The Nursing Database (BDENF), MedCarib and WHOLIS, Cumulative Index to Nursing and Allied Health Literature (CINAHL), CAPES Theses and Dissertations Catalog, and Google Scholar published and unpublished studies. A methodical manual search was also performed, including articles, editorials, and the references of the included studies, aiming to complete the search and identify any relevant studies not indexed in the databases.

The complete search strategy used in MEDLINE and adapted to the other electronic sources is shown in Table 1. The references of the included studies were analyzed for additional references of interest. There was no restriction regarding the scenario, place, date or language of publication.

**Table 1:** Search strategy used in MEDLINE and adapted to the other sources, according to selected descriptors.

Strategy	Descriptors used
# 1	(aged[tiab]) OR (“aged, 80 and over”[tiab]) OR (aging[tiab]) OR (older[tiab]) OR (elder[tiab]) OR (“older adults”[tiab]) OR (“oldest old”[tiab]) OR (“very old”[tiab]) OR (“very elderly”[tiab])
# 2	(psychometric*[tiab]) OR (“validation studies”[tiab]) OR (clinimetric*[tiab]) OR (“internal consistency” [tiab]) OR (tool[tiab]) OR (tools[tiab]) OR (instruments[tiab]) OR (instrument[tiab]) OR (screening[tiab) OR (“predictive value”[tiab]) OR (sensitivity[tiab]) OR (questionnaire[tiab]) OR (assessment[tiab]) OR (evaluation[tiab]) OR (“self-reported”[tiab]) OR (“self-report”[tiab]) OR (validity[tiab])
# 3	(“frail elderly”[tiab]) OR (“frailty elderly”[tiab]) OR (“frailty index”[tiab]) OR (“frailty syndrome”[tiab]) OR (“frail scale”[tiab]) OR (fragility[tiab]) OR (“pre-frailty”[tiab])
# 4	#1 AND #2 AND #3

The following inclusion criteria were applied: participants aged 60 years or over; studies describing an instrument capable of assessing frailty, and the presentation of clinimetric or cultural validation/adaptation properties of the frailty measurement instrument. Technical reports, letter to the editor, review articles and summary/annals of events were excluded. Two independent reviewers independently screened and selected the studies. Cases of disagreement were resolved by consensus. The data extraction was carried out by two independent reviewers using a pre-prepared form designed by the authors; disagreements were resolved by a third researcher.

The taxonomy and definitions used for the clinimetric properties evaluated followed criteria established by the COnsensus-based Standards for Health Measurement INstruments (COSMIN) [8] and were:

- Validity: refers to the extent to which an instrument measures the construct(s) for which it was constructed, including: content validity, construct validity, and criterion validity (concurrent validity, predictive validity).
- Reliability: highlights elements related to coherence, accuracy, stability, equivalence and homogeneity, i.e. principles to reproduce a result consistently in time and space, or from the perspective of different observers.
- Sensitivity: Probability of a positive test result if the subject tested presents the condition.
- Specificity: probability of a negative test result if the subject tested does not present the condition.
- Positive Predictive Value (PPV): defined as the proportion of true-positives among all individuals with positive test results.
- Negative Predictive Value (NPV): defined as the proportion of true-negatives among all individuals with negative test results.
- Cultural adaptation: adaptation of language and culture required when a scale or measure is used in a different country from that in which it was created and validated, to maintain the degree of performance of the items of the original version.

## Results

**Fig 1:**
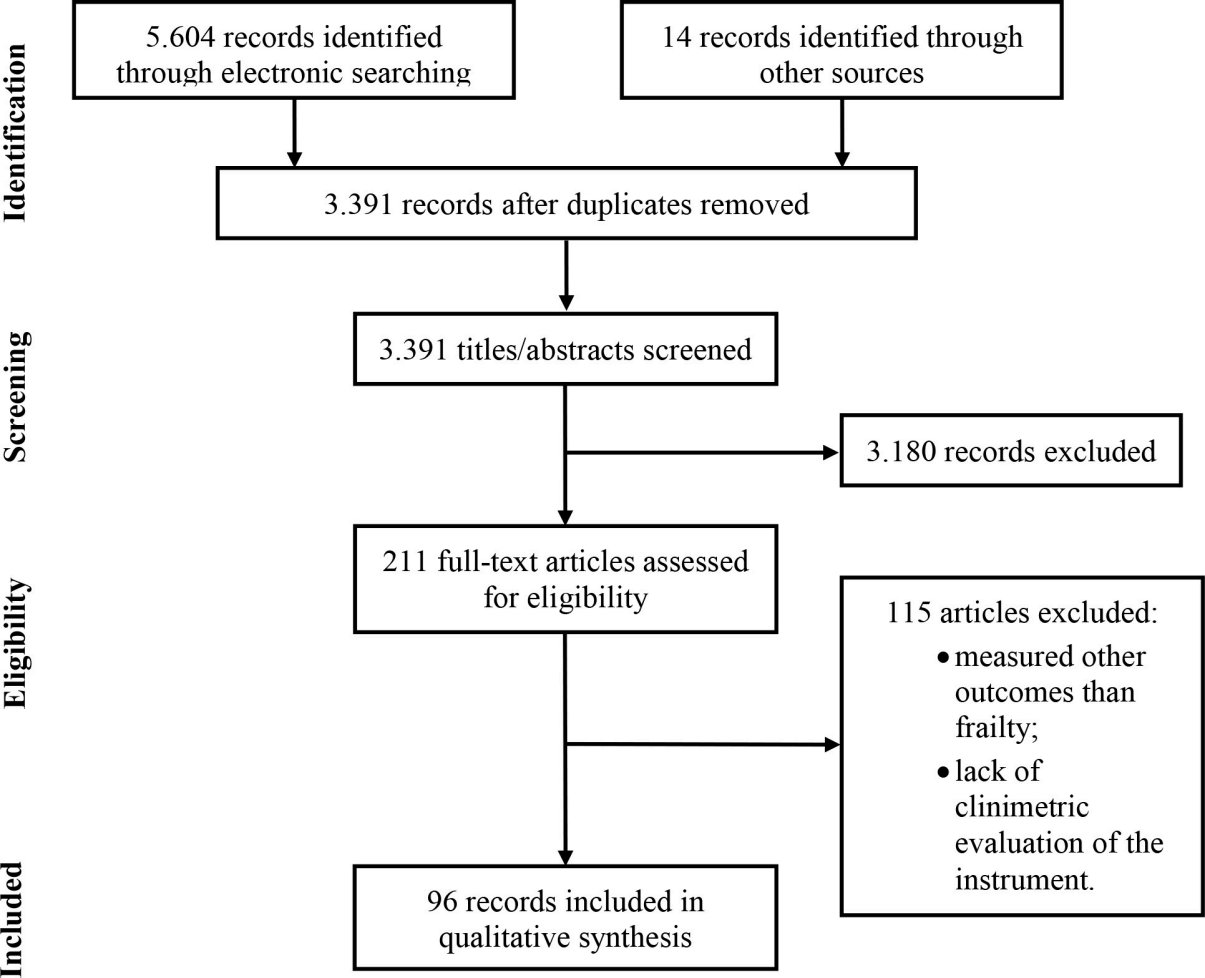
Study flow diagram.

The electronic searches returned a total of 5,604 records. After removing duplicates and including results of the handsearch (*n* = 14), 3,391 records remained, of which 3,180 were excluded based on title and abstract. The reading of the 211 remaining full-text publications led to the exclusion of 115 studies, since they included measures of constructs other than frailty (*n* = 66) or did not present information on the clinimetric evaluation of the instrument (*n* = 49). Accordingly, 96 studies met the pre-established criteria and were included in this review (Fig 1).

### Characteristics of the studies

A total of 51 frailty assessment instruments were analyzed in 96 studies published between 1997 and 2018. There were 82 studies published in English [18–99], 9 in Portuguese/BR [100–108], 3 in Japanese [109–111], 1 in Korean [112] and 1 in German [113]. The countries where the participants were most commonly sampled were the USA, with 14 studies [18, 21, 26, 49, 56, 61, 66, 70, 72, 77, 78, 86, 91, 95], Brazil, with 11 studies [64, 83, 92, 100–108], Canada, also with 11 studies [20, 22–24, 39, 54, 71, 80, 93, 94] and the Netherlands with 8 publications [28, 30, 32, 34, 36, 40, 55, 79, 96].

The instruments presented very heterogeneous characteristics, such as the number of items (3 to 92). Regarding the duration, some of the instruments were of rapid application (up to 10 minutes), while in some the evaluations are performed in more than one stage, which can last several hours. It should be noted that the majority of the publications did not report the application time [20, 22, 23, 29, 32, 36–38, 41–46, 49, 53, 55, 57–59, 63, 64, 66, 68, 69, 93, 97, 109–113].

Regarding the domains present in the frailty assessment instruments, 22 (11- point FI [77], 5-item mFI [95], Continuous Frailty Scale–CFS [91], electronic Frailty Index–eFI [58], Emergency General Surgeries Frailty Index–EGS-FI [61], FiND–Frail Non-Disabled [42], FRAIL Scale [53], Frailty Phenotype [70], Frailty Phenotype Modified [35], Frailty Screening Questionnaire (FSQ) [99], Frailty Trait Scale–FTS [44], *Instrumento Multidimensional de Rastreio da Síndrome da Fragilidade*–IMSIFI [101], INTER-FRAIL Study Questionnaire [43], LUCAS [113], *Modelo Fried adaptado* [106], Motor Performance Tests [64], PRISMA-7 [96], Self-Report Frailty Instrument [31], SHARE Frailty Instrument [74], SHARE Frailty Instrument 75+ [48], SOF Frailty Criteria [72], Trauma-Specific Frailty Index (TSFI) [78], UEF Frailty [56]) evaluated only physical aspects such as: slowness, weakness, inactivity, exhaustion, mobility, morbidities, activities of daily living (ADL), instrumental activities of daily living (IADL), functional capacity, signs and symptoms, laboratory exams, balance, gait, muscular strength, resistance, fatigue, physical activity, muscle mass index (MMI), sphincter control, weight loss, pain, falls, communication (vision, hearing), flexibility, hospitalization and use of medications.

A total of 9 instruments [18, 23, 27, 33, 45, 47, 73, 107, 112] assessed physical and psychological aspects (emotional aspects, such as mood alteration, motivation and reclusion), 1 [109] evaluated physical and social aspects (social support), 16 [19–21, 24, 25, 28–30, 39, 40, 49, 93, 94, 97, 98, 110] physical, psychological and social aspects, and 2, the Comprehensive Frailty Assessment Instrument (CFAI) [37, 38] and the Frailty GIR Evaluation (FRAGIRE) [68], used environmental indicators, as described by the authors, however, this aspect is also considered within the social domain and is defined by the social determinants of health, as indicated by the WHO [114], (housing conditions, comfort, stairs, distance to services and transport) in the frailty assessment component, as well as physical, psychological and social aspects (Table 2).

**Table 2:** Description of the instruments identified in the review and their characteristics: Number of items, domains, application scenario, language, study site, type of measurement scale, pre-frailty verification and mortality prediction.

**Instrument**	**Authors, Year**	**No. items**	**Domains**	**Settings**	**Language**	**Country**	Scale type*	**Pre-frailty**	**Mortality**
**11-point FI**	Velanovich et al., 2013	11	Ph	Hospital	English	USA	Dichotomous scale (frail—not frail) Range: 0–11	_	Yes
**5-item mFI**	Chimukangara et al., 2017	5	Ph	Hospital	English	USA	Dichotomous scale (frail—not frail) Range: 0–5	_	Yes
**68-item FI**	Ma et al., 2016	68	Ph, Ps, S	Community	English	China	Continuous Scale: 0–1. Combination of tests. ≥0,25 frail	_	Yes
**Brief Frailty Index**	Freiheit et al., 2010	5	Ph, Ps, S	Hospital	English	Canada	Dichotomous scale Frail—Not Frail ≥3 frail	_	Yes
**British frailty index**	Kamaruzzaman et al., 2010	35	Ph, Ps, S	Community	English	UK	Dichotomous scale (frail—not frail)	_	Yes
**Comprehensive Frailty Assessment Instrument–CFAI**	De Witte et al., 2013; De Witte et al., 2013	23	Ph, Ps, S, En	Community	English	Belgium, China	Dichotomous scale (frail—not frail) Range: 19–97. Does not have a cutoff point	_	No
**Instrument**	**Authors, Year**	**No. items**	**Domains**	**Settings**	**Language**	**Location of study**	Scale type*	**Pre-frailty**	**Outcome mortality**
**Clinical Global Impression of Change in Physical Frailty CGIC-PF**	Studenski et al., 2004	38	Ph, Ps, S	Community	English	USA	Dichotomous scale (frail—not frail)	_	No
**Continuous Frailty Scale–CFS**	Wu et al., 2018	5	Ph	Community	English	USA	Ordinal Scale: 3 levels. Range: 0–5, 0 Robust, 1–2 pre-frail, ≥3 frail	Yes	Yes
**CP-FI-CGA–Care Partners Frailty Index Comprehensive Geriatric Assessment**	Goldstein et al., 2013; Goldstein et al., 2015	62	Ph, Ps, S	Community, Emergency, Geriatric clinic	English	Canada	Dichotomous scale (frail—not frail)	_	Yes
**Clinical Frailty Scale–CSHA**	Rockwood et al., 2005; Gregorevic et al., 2016	70	Ph, Ps	CommunityHospital	English	Canada, Australia	Ordinal Scale: 1–7 7 levels (from robust to complete dependence)	Yes	Yes
**CSHA CFS TV—Chinese Canadian Study of Health and Aging Clinical Frailty Scale Telephone Version**	Chan et al., 2010	17	Ph, Ps	Community	English	Taiwan	Ordinal Scale: 1–7 7 levels (from robust to complete dependence). Phone version of the CSHA Clinical Frailty Scale.	Yes	Yes
**Instrument**	**Authors, Year**	**No. items**	**Domains**	**Settings**	**Language**	**Location of study**	Scale type*	**Pre-frailty**	**Outcome mortality**
**EASY-Care Two-step Older persons Screening—Easycare TOS**	Van Kempen et al., 2013; Van Kempen et al., 2014	38	Ph, Ps, S	Community	English	Nether-lands	Dichotomous scale (frail—not frail). Two-phase evaluation. 1^st^ phase—clinical reasoning, 2^nd^ phase—home evaluation	_	No
**Electronic Frailty Index–eFI**	Clegg et al., 2016	36	Ph	Community	English	UK	Ordinal Scale: 0–1 3 levels (robust, mild frailty, moderate frailty)	Yes	Yes
**Edmonton Frail Scale—EFS**	Rolfson et al., 2006; Fabrício-Wehbe et al., 2009; Fabrício-Wehbe, 2013; Ramírez et al., 2017	11	Ph, Ps, S	Community	English PT/BR	Canada, Brazil, Colombia	Ordinal Scale: 0–17 5 levels (not frail, apparently vulnerable, mild, moderate and severe frailty)	Yes	Yes
**Emergency General Surgeries Frailty Index–EGS-FI**	Jokar et al., 2016	15	Ph	Community	English	USA	Dichotomous scale (frail—not frail) Range: 0–1, >0.25—frail	_	Yes
**Frailty Index for Elders—FIFE**	Tocchi et al., 2014	10	Ph, Ps, S	Community	English	USA	Dichotomous scale (frail—not frail) Range: 0–10, >4 frail	_	No
**FiND—Frail Non-Disabled**	Cesari et al., 2014	5	Ph	Community	English	France	Dichotomous scale (frail—not frail) Separates disability from frailty	_	No
**Instrument**	**Authors, Year**	**No. items**	**Domains**	**Settings**	**Language**	**Location of study**	Scale type*	**Pre-frailty**	**Outcome mortality**
**FRAGIRE -Frailty GIR Evaluation**	Vernerey et al., 2016	19	Ph, Ps, S, En	Community	English	France	Continuous Scale: 0–100. There is no cut-off point. Higher scores equate to greater frailty	_	No
**FRAIL–Frailty and Autonomy Scoring Instrument of Leuven**	De Lepeleire et al., 2004	12	Ph, Ps, S	Community	English	Belgium	Dichotomous scale (frail—not frail) Range: 1–6. Does not have a cutoff point	_	No
**FRAIL Scale**	Gardiner et al., 2015; Woo et al., 2015; Gonzalez et al., 2016; Jung et al., 2016; Rosas-Carrasco et al., 2016; Aprahamian et al., 2017; Braun et al., 2018; Dong et al., 2018	5	Ph	Community	English	USA, Australia, China, South Korea, Mexico, Brazil, Germany	Ordinal Scale: 0–5 3 levels (not frail, pre-frail, frail). 0 Robust, 1 to 2 pre-frail, ≥3 frail	Yes	Yes
**Frailty Index (FI/CSHA)**	Mitnitski, 2001; Mitnitski et al., 2005; Widagdo et al., 2016; Abete et al., 2017	92	Ph, Ps, S	Community	English	Canada, Australia, Italy	Continuous Scale: 0–1. Combination of tests and self-report. Does not have a cutoff point	_	Yes
**Frailty Index (FI/CGA)**	Jones et al., 2004; Jones et al., 2005	_	Ph, Ps, S	Community, LTCIOA	English	Canada	Ordinal Scale: 0–20 3 levels (mild, moderate and severe frailty)	Yes	Yes
**Instrument**	**Authors, Year**	**No. items**	**Domains**	**Settings**	**Language**	**Location of study**	Scale type*	**Pre-frailty**	**Outcome mortality**
**Frailty Phenotype**	Fried et al., 2001; Kiely et al., 2009	5	Ph	Community	English	Australia, USA	Ordinal Scale: 0–5 3 levels (not frail, pre-frail, frail). ≥3 frail	Yes	Yes
**Frailty Phenotype Modified**	Saum et al., 2012	5	Ph	Community	English	Germany	Ordinal Scale: 0–5 3 levels (not frail, pre-frail, frail)	Yes	No
**Frailty Screening Questionnaire (FSQ)**	Ma et al., 2018	4	Ph	Community	English	China	Ordinal Scale: 0–4 3 levels (not frail, pre-frail, frail). ≥3 frail. Auto-relato	Yes	Yes
**Frailty Trait Scale—FTS**	Garcia-Garcia et al., 2014	12	Ph	Community	English	Spain	Ordinal Scale: 0–5 3 levels (not frail, pre-frail, frail)	Yes	Yes
**Geriatric Functional Evaluation (GFE)**	Scarcella et al., 2005	32	Ph, Ps, S	Community	English	Italy	Ordinal Scale: 3 levels (severely impaired, moderately impaired, totally independent)	Yes	Yes
**Gronigen Frailty Indicator–GFI**	Metzelthin et al., 2010; Daniels et al., 2012; Peters et al., 2012; Bielderman et al., 2013; Borges, 2013; Olaroiu et al., 2014; Peters et al., 2015; Braun et al., 2018	15	Ph, Ps, S	CommunityHospital LTCIOA	English, PT/BR	Nether-lands, Romania, Brazil, Germany	Dichotomous scale (frail—not frail). Range: 0–15. ≥4 frail	_	Yes
**Instrument**	**Authors, Year**	**No. items**	**Domains**	**Settings**	**Language**	**Location of study**	Scale type*	**Pre-frailty**	**Outcome mortality**
**Health Status Form–HSF**	Brody et al., 1997	16	Ph, Ps	Community	English	USA	Dichotomous scale (frail—not frail). Self-report screening instrument	_	No
**Instrumento Multidimensional de rastreio da Síndrome da Fragilidade–IMSIFI**	Lindôso, 2012	5	Ph	Community	PT/BR	Brazil	Ordinal Scale: 0–5 3 levels (not frail, pre-frail, frail)	Yes	No
**INTER-FRAIL Study Questionnaire**	Di Bari et al., 2014	10	Ph	Community	English	Italy	Dichotomous scale (frail—not frail)	_	No
***Índice de Vulnerabilidade Clínico-Funcional* IVCF-20**	Moraes et al., 2016	20	Ph, Ps	Community	PT/BR	Brazil	Ordinal Scale: 0–40 3 levels (robust, potentially frail, frail)	Yes	No
**Kaigo-Yobo Check-List**	Shinkai et al., 2010; Shinkai et al., 2013	15	Ph, Ps	Community	Japanese	Japan	Dichotomous scale (frail—not frail). Range: 0–15. >4 frail	_	No
**Klosha Frailty Index–KFI**	Jung et al., 2014	_	Ph, Ps	Community	English	South Korea	Dichotomous scale (frail—not frail) Range: 0–1	_	Yes
**Korean Frailty Index**	Hwang et al., 2010	8	Ph, Ps	LTCIOA	Korean	South Korea	Ordinal Scale: 3 levels (robust, pre-frail, frail)	Yes	No
**Instrument**	**Authors, Year**	**No. items**	**Domains**	**Settings**	**Language**	**Location of study**	Scale type*	**Pre-frailty**	**Outcome mortality**
**Kihon Check-List (KCL)**	Ogawa et al., 2011; Sampaio et al., 2014; Satake et al., 2016	25	Ph, Ps, S	Community	Japanese, English, PT/BR	Japan, Brazil	Dichotomous scale (frail—not frail) Range: 0–25. Does not have a cutoff point	_	No
**LUCAS**	Dapp et al., 2012	12	Ph	Community	German	Germany	Ordinal Scale: 0–6 3 levels (healthy, pre-frail, frail)	Yes	No
**Mini-Nutritional Assessment MNA-SF**	Dent et al., 2012	14	Ph, Ps	Hospital	English	Australia	Dichotomous scale (frail—not frail) Range: 0–14. <9 frail	_	No
***Modelo Fried adaptado***	Nunes et al., 2015	6	Ph	Community	PT/BR	Brazil	Ordinal Scale: 0–5 3 levels (not frail, pre-frail, frail), ≥3 frail	Yes	No
**Motor Performance Tests**	Santos et al., 2016	2	Ph	Community	English	Brazil	Dichotomous scale (frail—not frail)	_	No
**PRISMA-7**	Raîche et al., 2008; Saenger et al., 2016; Braun et al., 2018;	7	Ph	Community	English, PT/BR	Canada, Brazil, Germany	Dichotomous scale (frail—not frail) Range: 0–7, ≥3 frail	_	No
**Prognostic Frailty Score**	Ravaglia et al., 2008; Widagdo et al., 2016	9	Ph, Ps	Community	English	Italy, Australia	Continuous Scale: 0–9. Does not have a cutoff point Self-report and performance test	_	Yes
**Instrument**	**Authors, Year**	**No. items**	**Domains**	**Settings**	**Language**	**Location of study**	Scale type*	**Pre-frailty**	**Outcome mortality**
**SEGAm–Modified Short Emergency Geriatric Assessment**	Oubaya et al., 2014	13	Ph, Ps	Community	English	France	Ordinal Scale: 0–13 3 levels (mild, moderate and severe frailty)	_	No
**Reported Edmonton Frail Scale–REFS**	Hilmer et al., 2009	8	Ph, Ps, S	Hospital	English	Australia	Ordinal Scale: 0–18. 5 levels (not frail, apparently vulnerable, mild, moderate and severe frailty). Adapted version of the Edmonton Frail Scale	Yes	No
**Self-Report Frailty Instrument**	Barreto et al., 2012	4	Ph	Community	English	France	Ordinal Scale: 0–4 3 levels (healthy, pre-frail, frail)	Yes	Yes
**SHARE Frailty Instrument**	Romero-Ortuno et al., 2010; Romero-Ortuno et al., 2013	5	Ph	Community	English	Multicenter	Ordinal Scale: 3 levels (not frail, pre-frail, frail)	Yes	Yes
**SHARE Frailty Instrument 75+**	Romero-Ortuno et al., 2014	4	Ph	Community	English	Multicenter	Ordinal Scale: 3 levels (not frail, pre-frail, frail)	Yes	Yes
**SOF Frailty Criteria**	Ensrud et al., 2008; Kiely et al., 2009; Bilotta et al., 2012	3	Ph	Community	English	USA, Australia, Italy	Ordinal Scale: 3 levels (not frail, pre-frail, frail)	Yes	Yes
**Trauma-Specific Frailty Index (TSFI)**	Joseph et al., 2014	15	Ph	Hospital	English	USA	Dichotomous scale (frail—not frail), >0.27 frail	_	No
**Instrument**	**Authors, Year**	**No. items**	**Domains**	**Settings**	**Language**	**Location of study**	Scale type*	**Pre-frailty**	**Outcome mortality**
**UEF Frailty**	Toosizadeh et al., 2015; Toosizadeh et al., 2016; Toosizadeh et al., 2017	8	Ph	Community, Hospital	English	USA	Ordinal Scale: 3 levels (not frail, pre-frail, frail)	Yes	No
**Tilburg Frailty Indicator–TFI**	Gobbens et al., 2010; Metzelthin et al., 2010; Daniels et al., 2012; Santiago, 2013; Santiago et al., 2013; Andreasen et al., 2014; Uchmanowicz et al., 2014; Andreasen et al., 2015; Coelho et al., 2015; Freitag et al., 2016; Uchmanowicz et al., 2016; Mulasso et al, 2016; Dong et al., 2017; Vrotsou et al., 2018	15	Ph, Ps, S	Community, Hospital, LTCIOA	English, PT/BR	Nether-lands, Denmark, Poland, Portugal, Germany, Brazil, Italy, China, Spain	Dichotomous scale (frail—not frail). Range: 0–15, ≥5 frail	_	Yes

*Scale: ordinal, continuous, dichotomous; PT: Portuguese; Ps: Psychological; Ph: Physical; S: Social; En: Environmental

Regarding the application scenario, of the 51 instruments identified [18–21, 23–25, 27–31, 33, 35, 37, 39, 40, 42–45, 47–49, 53, 56, 58, 61, 64, 68, 70, 72–74, 77, 78, 91, 93–99, 101, 106, 107, 109, 110, 112, 113], 38 [18, 19, 21, 24, 27, 29, 31, 35, 37, 40, 42–45, 47–49, 53, 58, 61, 64, 68, 70, 72–74, 91, 93, 96–99, 101, 106, 107, 109, 110, 113] were constructed and/or validated for use with the older adult population in the community context, 6 [25, 33, 77, 78, 91, 94, 95] were only validated for use in the clinical context, and 7 instruments [20, 23, 28, 30, 39, 56, 112] were validated for both contexts, including Long-Term Care Institutions for Older Adults (LTCIOA).

### Ability to identify pre-frailty

A total of 23 instruments presented three to six levels of frailty [20, 23–25, 27, 31, 35, 44, 48, 53, 56, 58, 70, 72, 74, 91, 98, 99, 101, 106, 107, 112, 113]. These levels classified the older adult participants as follows: robust or not frail, pre-frail or apparently vulnerable, mild frailty, moderate frailty and severe frailty, using a numerical score.

### Ability to predict mortality

Mortality is an adverse health outcome and is associated with frailty. In this review, 27 frailty evaluation instruments with the ability to predict mortality were identified [20, 23, 27, 29, 31, 32, 44, 45, 48, 54, 58, 59, 61, 70, 72–74, 77, 85, 91, 93–95, 97–99].

### Clinimetric properties

The instruments with the highest number of clinimetric properties evaluated were the FRAIL Scale and the Edmonton Frail Scale–EFS, in which nine domains were evaluated, with the FRAIL Scale having been culturally adapted in seven countries. The FRAGIRE and IVCF-20 had eight domains evaluated. The GFI and TFI had seven items evaluated, with versions having already been developed in 49 countries, with their adaption to the languages and cultures. In contrast, the 11-point FI [77], 5-item mFI [95], Continuous Frailty Scale [91], Emergency General Surgeries Frailty Index [61], Frailty Phenotype Modified [35], Frailty Screening Questionnaire (FSQ) [99], Geriatric Functional Evaluation (GFE) [98], Klosha Frailty Index [45] and the LUCAS [113] had only one measure attribute evaluated. Table 3 provides an overview of the measurement properties of each frailty assessment instrument.

**Table 3:** Frailty assessment instruments and their clinimetric properties.

**Instruments**	**Reliability**			**Validity**				**Other Attributes**		
	**Internal Consistency**	**Equivalence**	**Stability**	**Content Validity**	**Construct Validity**	**Criterion Validity**	**Cross-cultural Validity**	**Sensitivity**	**Specificity**	**PPV/ NPV**
**11-point FI** [77]	●									
**5-item mFI** [95]						●				
**68-item FI** [97]					●	●				
**Brief Frailty Index** [94]				●		●				
**British frailty index** [29]					●		●	●		
**CFAI** [37, 38]	●	●			●	●	●			
**CGIC-PF** [21]		●	●	●						
**CFS** [91]					●					
**CP-FI-CGA** [39, 54]				●	●	●				
**Clinical Frailty Scale–CSHA** [23, 60]		●	●		●	●	●			
**CSHA CFS TV** [27]		●	●			●				
**Easycare TOS** [40, 79]		●			●	●				
**eFI** [58]					●	●	●			
**EFS** [24, 85, 100, 103]	●	●	●	●	●	●	●	●	●	
**EGS-FI** [61]						●				
**FIFE** [49]	●			●						
**FiND** [42]								●	●	
**FRAGIRE** [68]	●		●	●	●	●		●	●	●
**FRAIL** [19]	●			●				●	●	●
**FRAIL Scale** [53, 57, 59, 62, 63, 87]	●	●		●	●	●	●	●	●	●
**Instruments**	**Reliability**			**Validity**				**Other Attributes**		
	**Internal Consistency**	**Equivalence**	**Stability**	**Content Validity**	**Construct Validity**	**Criterion Validity**	**Cross-cultural Validity**	**Sensitivity**	**Specificity**	**PPV/ NPV**
**Frailty Index (FI/CGA)** [20, 22]		●	●	●	●	●	●	●		
**Frailty Index (FI/CSHA)** [69, 71, 93]	●				●			●		
**Frailty Phenotype** [70]					●	●	●			
**Frailty Phenotype Modified** [35]					●					
**FSQ** [99]						●				
**Frailty Trait Scale–FTS** [44]					●	●		●	●	●
**Geriatric Functional Evaluation (GFE)** [98]					●					
**GFI** [30, 32, 34, 36, 46, 55, 87, 102]	●			●	●	●	●	●	●	●
**HSF** [18]		●			●			●	●	●
**IMSIFI** [101]					●	●		●	●	●
**INTER-FRAIL Study Questionnaire** [43]						●		●	●	●
**IVCF-20** [107]	●	●		●	●	●		●	●	●
**Kaigo-Yobo Check-List** [109, 111]	●				●	●		●	●	
**KFI** [45]						●				
**KCL** [65, 92, 110]	●				●	●	●	●	●	
**Korean Frailty Index** [112]	●							●	●	●
**LUCAS** [113]				●						
**MNA-SF** [33]						●		●	●	●
***Modelo Fried adaptado*** [106]	●					●		●	●	●
**Motor Performance Tests** [64]						●		●	●	
**Instruments**	**Reliability**			**Validity**				**Other Attributes**		
	**Internal Consistency**	**Equivalence**	**Stability**	**Content Validity**	**Construct Validity**	**Criterion Validity**	**Cross-cultural Validity**	**Sensitivity**	**Specificity**	**PPV/ NPV**
**PRISMA-7** [87, 96, 108]				●	●	●	●	●	●	●
**Prognostic Frailty Score** [69, 73]					●		●			
**REFS** [25]	●	●			●					
**SEGAm–Modified Short Emergency Geriatric Assessment** [47]	●		●		●					
**Self-Report Frailty Instrument** [31]					●	●				
**SHARE Frailty Instrument** [74, 76]	●			●	●	●	●			
**SHARE Frailty Instrument 75+** [48]	●				●	●	●			
**SOF Frailty Criteria** [26, 69, 72, 75]					●	●	●			
**TFI** [28, 30, 32, 41, 50–52, 67, 80, 104, 105]	●		●	●	●	●	●	●	●	
**TSFI** [78]								●	●	
**UEF Frailty** [56, 66, 86]					●			●	●	

● Instrument fulfills the criteria mentioned

PPV, Positive Predictive Value; NPV, Negative Predictive Value.

## Discussion

In this review, 51 instruments that tested for frailty in older adults were presented. The domains that constituted these instruments were predominantly physical; however, elements of a psychological, social and environmental order were observed in the instruments developed more recently. Using broader approaches is one of the points of relevance in the context of frailty, since the exclusive focus on physical problems can lead to the fragmentation of care for older adults [28]. The association between frailty and social factors has been widely recognized, with social isolation also being significantly associated with mortality. Social relations play a central role in human well-being and are directly involved in maintaining health [115].

The different domains used for the construction of the instruments follow the concept of frailty adopted by each researcher, although the concept of frailty is currently consensual among researchers and the clinical aspect prevails [116]. However, the same does not apply to the evaluation criteria [117] and from this perspective, the investigation of the accuracy of the frailty measures for the prediction of adverse health events has gained space, while the validity and reliability of the frailty measures reveal a gap in the literature [118].

The CFAI instrument, developed based on data from the Belgian Study of Aging, and the FRAGIRE include the environmental component in the multidimensional assessment of frailty and exclude the disability and comorbidities items. The CFAI also allows the evaluation of frailty through postal and telephone interviews [37], unlike the FRAGIRE, which is administered by a trained interviewer.

Precariousness of the housing situation and conditions such as reduced space, lack of physical facilities and barriers to housing and services increase the risk of vulnerability to stressors and have been related to frailty [119, 120]. Many older adults remain in their own homes for as long as possible, due to the possibility of greater autonomy when compared to aging in nursing homes and the favorable psychosocial aspects of remaining in the same environment. However, older adults with higher incomes and better health opt for care in institutions [121]. Therefore, including environmental conditions in the assessment of frailty among older adults is advisable.

In the clinical setting, frailty assumes unquestionable importance, with the current challenge being to operationalize the concept and facilitate its recognition [116]. There are several scenarios in which the measurement of frailty can be performed using different instruments, such as in primary care [11], emergency units/departments, general hospitals, long-term care facilities and nursing homes. Despite being a progressive condition, frailty can be prevented and rehabilitated, and therefore, in terms of public health, instruments designed to identify frail older adults living in the community, as was the case for the majority in this review, allow early intervention and management of risk factors. This contributes to prioritize approaches with older adults with frailty already installed and opens a series of possibilities for individual or collective actions also among non-frail older adults [122].

In this context, the instruments that identify pre-frailty present positive aspects. When the syndrome and/or its risk factors are diagnosed early, the disability resulting from frailty can be better treated and the prognosis will be more positive, i.e., interventions are more effective when applied with older adults in the initial stage of frailty [123, 124]. Although frailty is a dynamic process, characterized by frequent transitions over time, the probability of transition to states of greater frailty is greater than the transitions to states of lower frailty, and the chance of transition from “very frail” to a robust status is extremely low, even over long periods [125].

Various ways of measuring this construct were found in the literature, identifying self-administered questionnaire, questionnaires or interviews, performance tests and combinations of these. The choice can be made according to the different scenarios (hospital, primary care, long term care), the aim of the measurement, the qualification (physician, general practitioner, nurse, caregiver) of the interviewer and the time available. Each instrument has advantages and disadvantages, so that, in the composition of the sample in relation to age and nationality, it is important to compare the results measured by these instruments with each other [9].

A predominance of instruments based on the Phenotypic Frailty Model and the Cumulative Deficit of the CSHA was observed. Three instruments adapted from the Phenotypic model, the Frailty Phenotype Modified [35], *Modelo Fried adaptado* [106] and CFS [91], were developed to overcome the limitations of the original instrument. One of these is the use of measured variables with dichotomous criteria. In addition, all the indicators of the scale are considered of equal importance in the measurement of frailty and effective in identifying the most frail older adults [126]. Also, the measurement of some components of the syndrome requires specialized equipment and/or training, which makes it difficult to use in primary care [106].

More important than the ideal instrument, the aspect that really should be considered is the common aim of the different actors involved, i.e. whether the focus is to carry out screening or evaluation, as these have different characteristics due to their different levels of complexity. Screening instruments are different to evaluation instruments, with it being possible to perform these procedures in two steps (first step: multidimensional screening, for all individuals and; second step: evaluation only for the frail) [127]. Screening instruments for application in PHC should be of short duration, if possible, administered by telephone and by different professionals (physician, general practitioner, nurse), in order to easily reach a large number of individuals and still be accurate concerning negative adverse results [128].

The *Modelo Fried adaptado* is a self-referenced instrument, which allows the expansion of screening for the syndrome, as well as serving as a “sentinel” in its early identification [106]. The CFS became a continuous scale, with high agreement with the original scale, and identified that gait speed and weight loss were the strongest and weakest indicators, respectively [91]. The gait speed is a rapid, inexpensive and easy to assess physical performance measure, integrating the health assessment and a well documented risk factor for adverse outcomes in older adults. Weight loss, verified in two visits at least one year apart, may be more susceptible to measurement errors than the other indicators, which may explain the fact that it is the weakest indicator of frailty.

All the instruments identified in this review demonstrate evidence supporting the robustness of these models; however, studies on the reliability or validity of the original versions of many of the instruments are still scarce. Measuring instruments must have certain characteristics which ensure the reliability of the data produced [129].

Guidelines that describe basic principles for instrument construction recommend the performance of at least reliability and validity tests [7]. In this review, the choice was made to list the dimensions explicitly cited by the authors, not allowing interpretations of data that could only suggest tests performed. Criterion validity, which integrates the predictive and concurrent validity [8], was considered when either was cited. For example, the Frailty Phenotype (70) provided predictive validity, when it evaluated the association, prospectively, with five important adverse health outcomes found in the 4 and 7 year prospective follow-up, using Cox proportional hazards models, with data of the Cardiovascular Health Study.

The FRAGIRE [68], FRAIL Scale [53], EFS [24] and IVCF-20 [107] instruments were the most frequently examined for clinimetric properties, which were not mentioned in the most recent systematic review of the literature [2]. Likewise, the TFI presents very solid statistical results, with it having been used in studies with large samples [11], also due to its clinimetric qualities evaluated [2]. Validated in six countries, it is multidimensional and can be applied in the community, in the hospital setting and in LTCIOA and does not include variables that are considered frailty outcomes, such as disability, falls and hospitalization. However, Vrotsou et al. [90] recommend additional studies in different social contexts, as the different social realities in Europe and the rest of the world do not seem to have been contemplated in designing and validating the scale and therefore its applicability at different stages of frailty should be reconsidered.

Accordingly, this review provides a broad overview of the instruments proposed for assessing frailty in older adults over the past 20 years, which are based on two main approaches: unidimensional, related to physical health, and multidimensional, which includes psychological, social and, more recently, environmental aspects. None of the 51 instruments analyzed were examined for reliability and validity in relation to all the domains. Furthermore, some instruments require validation in larger studies and, therefore, it is difficult to highlight which instrument, at present, is the best for the screening of frailty in older adults. It should be noted that clinical judgment is still the best tool available to evaluate the individual needs of a patient. In addition, special attention should be paid to common problems of advanced age, such as the reduction of economic resources, reduced mobility and loss of loved ones, which contribute to limiting social contact [115], with socially isolated individuals presenting an increased risk for the development of cardiovascular diseases [130] and cognitive decline [131].

Furthermore, it was noted that each instrument defined an interviewer (physician, nurse, team), a minimum age (60 years, 70 years, 75 years), a short or long application time and mobilized a scientific community in search of a definition for frailty and of a robust instrument capable of measuring it and screening for it.

The ability to perceive the mutability of frailty over time and the interaction of physical, psychological, social and environmental domains should be part of the ability of trained and conscientious professionals in the care for older adults as part of the comprehension of the dynamic and complex system of the aging process. Thus, investing in health teams so that they are able to recognize frailty in different areas raises other important lines as a basis for future studies. In addition, concerning the implications for new studies, the need for standardization of the scales is emphasized, since the use of different instruments in clinical trials may prevent the comparability of the results in systematic reviews. Due to different instruments and applicability scenarios, the possibility of comparing studies constitutes an important step.

Healthcare providers must consider that the process of identifying frailty should be based on a simple test, requiring little time and few resources, which can be interpreted by non-specialist professionals. Accordingly, they must, among the various instruments identified in this review, opt for the one that is translated and validated for their location and that shows itself to be the most adequate for their context.

## Strengths and limitations

The strengths of this review include the comprehensive electronic search of 14 sources, with no limitations regarding language or date of publication, as well as the manual search in the references of the included studies. In addition, as far as is known, this review was the first to present a broad view of instruments that detect frailty, with information that includes the domains, population, setting, type of scale, outcome mortality and clinimetric properties. This contributes so that professionals and researchers can make a better choice of the instrument, specific to the scenario and the scope of each study.

In addition, this review avoided applying exclusion criteria, unlike previous studies that restricted: (I) the age, studying only individuals aged 65 years and over [10, 13], which limits the external validity of the studies, since in developing countries, according to the World Health Organization (WHO), older adults are those that are 60 years of age or more; (II) the scenario, evaluating only the non-hospitalized population [11, 13], restricting the external validity of the study; and (III) the language, favoring publication and selection bias [2, 10, 132].

One limitation found in this study was the confusion among the scales, because sometimes a specific instrument is named differently in different studies. In addition, the risk inherent in any systematic review of not having located all the relevant studies was recognized, despite the methodological rigor and care taken by the authors for this not to occur.

## Supporting information

S1 PRISMA Checklist (DOC)
